# Developing expertise in laparoscopic management of total colonic aganglionosis cases: Duhamel vs. Swenson procedures

**DOI:** 10.3389/fsurg.2026.1882822

**Published:** 2026-09-02

**Authors:** Ahmed Arafa, Mohamed Hamed Abouelfadl, Belal Eldomiaty, Aboubakr Ahmed, Ahmed Azzam, Mohamad Qinawy, Seham Anwar, Ahmed S. Ragab

**Affiliations:** 1 Pediatric Surgery Department, Faculty of Medicine, Cairo University, Cairo, Egypt; 2 Pediatric Surgery Department, Faculty of Medicine, Beni Suef University, Beni Suef, Egypt; 3 Pediatric Surgery Department, Faculty of Medicine, Portsaid University, Port Said, Egypt

**Keywords:** Duhamel, minimal invasive surgery, surgical expertise, Swenson, total colonic aganglionosis

## Abstract

**Aim:**

To describe technical evolution of minimally invasive surgery (MIS) for total colonic aganglionosis (TCA) either Duhamel or Swenson procedures conducted by a single pediatric surgical team.

**Methods:**

18 children with histologically proven TCA were managed between March 2018 and March 2022. All underwent laparoscopic definitive pull-through: Group A (*n* = 8) received laparoscopic-assisted transanal Swenson-type ileoanal pull-through, and Group B (*n* = 10) underwent laparoscopic Duhamel procedure. Thirteen patients presented with a pre-existing ileostomy; five were primary cases. Pre-operative evaluation included contrast enema and full-thickness rectal biopsy in every patient.

**Results:**

Median age at definitive surgery was 3 years (range 18 months–5 years; mean ± SD = 3.0 ± 0.9 years). Median operative time was 4 h (range 3.5–5.5 h) for Group A and 5 h (range 4.5–6 h) for Group B. Median follow-up was 5 years (range 3–6 years). Early complications included two anastomotic stenoses (Group A) successfully treated with dilatation. No anastomotic leaks occurred. Median daily stool frequency at 6 months was 7 (range 5–10) in Group A vs. 5 (range 4–7) in Group B, improving to 3 (range 2–4) and 2 (range 1–3), respectively, by last follow-up. Fecal incontinence occurred in two patients (Group A). Three patients required loperamide and six cases used dietary fiber modification.

**Conclusion:**

Laparoscopic management of TCA is safe and feasible. Both Swenson-type and Duhamel approaches yield acceptable continence and growth outcomes, with the choice tailored to rectal anatomy and continence risk.

## Introduction

Total colonic aganglionosis (TCA) is a rare and severe variant of Hirschsprung disease, accounting for roughly 3%–10% of all cases (16). It is characterized by aganglionosis extending from the anus up to 30–50 cm proximal to the ileocecal valve (1, 2). The primary surgical goal is complete removal of the aganglionic intestine and restoration of intestinal continuity with good long-term function.

Several operative strategies have been described, including Swenson, Duhamel, Soave, and Martin procedures (3–7, 15). Meta-analyses have not demonstrated clear superiority of one technique over another. The advent of minimally invasive surgery (MIS) has provided additional benefits such as superior visualization, improved cosmesis, reduced postoperative pain, and shorter recovery, easier dissection of proximal colon by laparoscope instead of midline incision, or to overcome difficulty in dissection of the proximal colon from Pfannenstiel incision especially if young children (8).

We report our single-team technical evolution during institutional experience with MIS for TCA, comparing laparoscopic-assisted transanal pull-through and laparoscopic Duhamel techniques, and present detailed functional outcomes after a minimum 3-year follow-up.

## Methods

A retrospective study was conducted from March 2018 to March 2022, 18 consecutive children with TCA were managed at our hospitals (cairo -benyswef -portsaid). All definitive pull-through procedures were initiated laparoscopically without initial open exploration. The timing of definitive surgery was (range: 18 months–5 years) for several causes: the age at presentation, clinical compensation, and optimization of nutritional status, especially for late-referral primary cases who were managed conservatively until toddlerhood.

This age range represents the most common period for definitive reconstruction after adequate bowel adaptation and allows reliable postoperative functional assessment due to further maturation of bowel function and continence mechanisms as toilet training and bowel control begins to develop around 3 years of age.

The choice of the procedure evolved throughout the study period as surgical experience accumulated and technical preferences developed. Allocation was not randomized and was influenced by surgeon judgment, patient specific anatomical considerations, evolving institutional practice. Patient allocation was strictly based on preoperative and intraoperative anatomical findings: Group A (*n* = 8) underwent laparoscopic-assisted transanal Swenson pull-through, which was selected for those with narrow distal rectum which to facilitate safe circumferential transanal dissection. Group B (*n* = 10) laparoscopic Duhamel procedure was done as a dilated proximal segment or a short rectal stump was structurally advantageous to perform a secure side-to-side stapled anastomosis and minimize pelvic floor dissection.

## Statistical analysis

Statistical analysis was performed in Table 1 using SPSS version 22.0 (IBM Corp., Armonk, NY, USA). Continuous variables, specifically the operative times, were expressed as medians with interquartile ranges (IQR) and compared between the two surgical groups using the non-parametric Mann–Whitney U test due to the small sample size. Categorical variables, including the rates of postoperative complications (fecal incontinence and enterocolitis), were expressed as frequencies and percentages and compared using Fisher's exact test. For all analyses, a two-sided *p*-value of less than 0.05 was considered statistically significant.

**Table 1:** Comparison between group A and group B.

Variable	Group A (Laparoscopic Swenson) (*n* = 8)	Group B (Laparoscopic Duhamel) (*n* = 10)	*p*-value (Statistical Test)	Significance (*p* < 0.05)
Median Operative Time (IQR)	4 h (3.5–5.5)	5 h (4.5–6)	*p* = 0.023 (Mann–Whitney U)	Yes (Statistically Significant)
Fecal Incontinence	2 (25%)	0 (0%)	*p* = 0.183 (Fisher's Exact)	No
Post-op Enterocolitis	1 (12.5%)	3 (30%)	*p* = 0.588 (Fisher's Exact)	No
Leakage	0 (0%)	0(0%)	*p* = 0.999 Fisher`s	No
Stenosis	2 (25%)	0(0%)	*p* = 0.183 Fisher`s	No

All patients underwent contrast (Gastrografin) enema to delineate the transition zone and full-thickness rectal biopsy confirming aganglionosis. Patients with major cardiac anomalies or total intestinal aganglionosis were excluded. intraoperative frozen-section biopsies were routinely performed at the proximal resection margin to ensure the presence of ganglion cells and the absence of hypertrophic nerve bundles before performing the anastomosis. Donut biopsies from the stapler or resected margins were fixed for permanent histopathology in all cases.

Group A (*n* = 8) underwent laparoscopic-assisted transanal Swenson pull-through, selected for patients without severe distal rectal dilatation. Group B (*n* = 10) underwent laparoscopic Duhamel procedure when a short rectal stump was advantageous.

All definitive pull-through procedures were initiated laparoscopically without need for initial open exploration. Technical refinements learned over time included low-pressure pneumoperitoneum (8–10 mm Hg) to reduce hypercapnia and stepwise vascular division to minimize bleeding.‏

The five primary TCA patients who presented at approximately 3 years of age were late referrals who had been managed conservatively with meticulous nursing care, adequate hydration, recurrent rectal irrigation and close monitoring. Conservative treatment was successful for previous history of obstruction or enterocolitis. This delayed presentation—although atypical—was documented in resource-variable settings where neonatal obstruction may be mild and partially compensated, allowing patients to reach toddler age before definitive diagnosis. This explains why safe primary pull-through was feasible in these cases.

## Operative procedure

Each operation consisted of a laparoscopic and a transanal component. The surgical team stood on the patient's right side to dissect the rectum, sigmoid, and descending colon. The table was tilted toward the surgeon as needed. To dissect the transverse colon, the surgeon moved to the patient's left side. Dissection continued to the cecum using a a bipolar vessel-sealing device (LigaSure™) device while carefully avoiding stomach injury. Pneumoperitoneum was maintained at 10 mm Hg with a flow rate of 2 L/min.

## Port placement

A camera port was inserted at the umbilicus plus four working ports: one in the lower right quadrant, one in the lower left quadrant, and one beneath the xiphisternum.

### Laparoscopic phase

**Figure 1:**
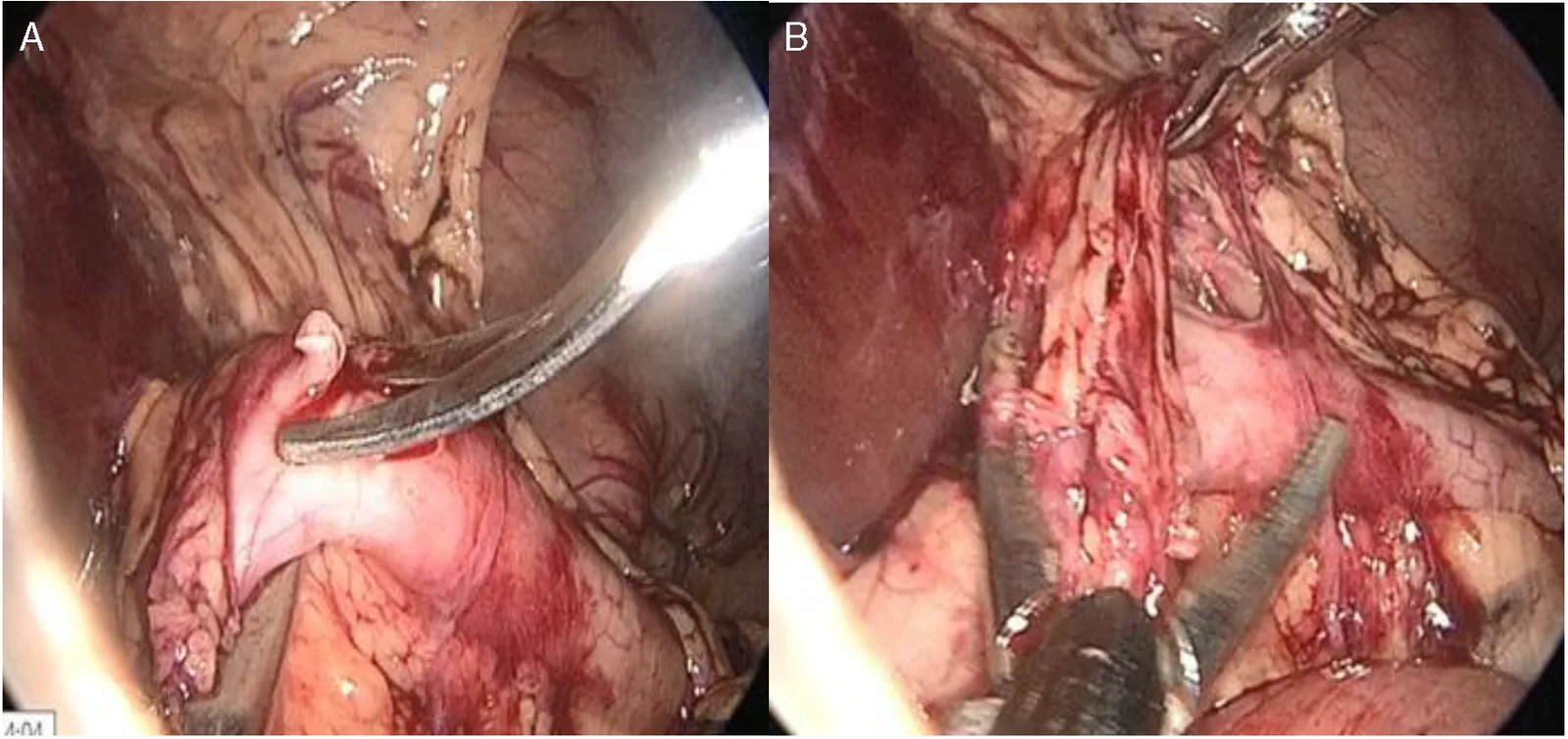
Laparoscopic preparatory phase for total colonic aganglionosis. **(A)**: Laparoscopic seromuscular biopsy of the transverse colon to confirm absence of ganglion cells. **(B)**: stepwise laparoscopic mobilization and dissection of transverse colon.

**Figure 2:**
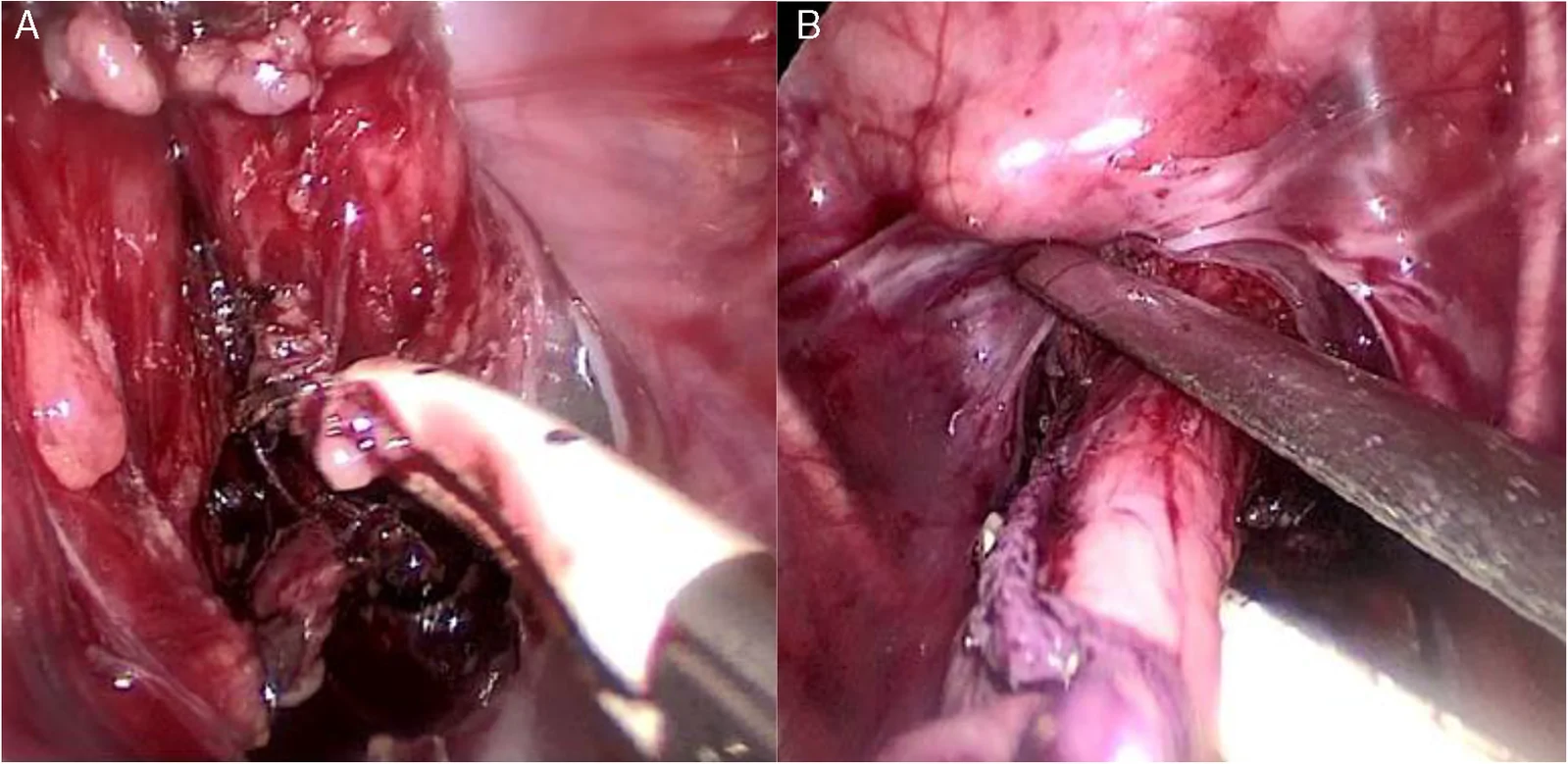
Laparoscopic steps of duhamel procedure: **(A)** dissection of the retrorectal avascular space during laparoscopic duhamel procedure. **(B)** low division of the aganglionic rectum using a 55 mm linear cutter gastrointestinal stapler to ensure a minimal rectal stump.

- Sequential seromuscular colonic biopsies were taken in an ascending fashion to confirm the absence of ganglion cells before resection.
- Complete resection of the aganglionic colon was conducted by stepwise devascularization of the left, middle, and right colic vessels (Figure 1).
- The peritoneal reflection was incised.
  - Group A: full circumferential mobilization of the aganglionic rectum.
  - Group B: only posterior mobilization on the retrorectal avascular plane, 1 cm above the dentate line (Figure 2A).
- In Group B, the aganglionic rectum was divided as low as feasible to leave a very short rectal stump, using a 55 mm linear gastrointestinal stapler (Figure 2B).

### Transanal phase

- Group A: A circular incision was made 1 cm above the dentate line; the rectum was dissected 2–3 cm from the anorectal wall and the entire colon removed.
- Group B: A lower semicircular incision 1 cm above the dentate line was made; the common rectal wall edges were held with stay sutures and anastomosed to the anterior ileal wall after pull-through, using a linear GI stapler (75 mm) and completing the ileoanal anastomosis with 4-0 Vicryl.

A final laparoscopic inspection confirmed proper orientation of the pulled-through ileum and the absence of twisting or intra-abdominal bleeding.

## Results

Age at surgery ranged 18 months–5 years (mean ± SD = 3.0 ± 0.9 years; median 3 years). Thirteen presented with an ileostomy created elsewhere for obstruction or intractable enterocolitis; five had no stoma (Table 2).

**Table 2:** Patient demographics and pre-operative status (*n* = 18).

Variable	Value
Age at definitive surgery	18 mo–5 yr (mean 3.0 ± 0.9 yr)
Male: Female	10: 8
Pre-existing ileostomy	13
Primary (no stoma)	5
Presenting symptoms	Chronic constipation (15), enterocolitis (6), obstruction (4)

In the 13 patients presenting with a pre-existing ileostomy, we didn't close the stoma at the the time of definitive pull through surgery to protect the newly constructed anastomosis, especially given the high stool frequency and potential perineal excoriation associated with TCA. Stoma closure was performed as a staged procedure 2–3 months later, after ensuring anastomotic healing and adequacy anal size diameter.

Median operative time was 4 h (range 3.5–5.5) for Group A and 5 h (range 4.5–6) for Group B. No intra-operative conversions were required. In our cohort, there were no cases where postoperative permanent histology demonstrated an aganglionic transition zone at the anastomosis, confirming successful clearance during the laparoscopic phase. Early complications included two cases of anastomotic stenosis (Group A) successfully managed with dilatation. No anastomotic leaks occurred.

Median follow-up was 5 years (range 3–6 years), representing a medium-term assessment. Early complications were observed: enterocolitis in 1 Group A patient and 3 Group B patients; constipation in 2 Group B patients; fecal incontinence in 2 Group A patients (Krickenbeck grade II); and perianal dermatitis in 3 Group A patients during the first postoperative year. Electrolyte imbalance due to frequent stools occurred in 1 Group A patient.

Median daily stool frequency declined from 7 (range 5–10) at 6 months to 3 (range 2–4) at last follow-up in Group A, and from 5 (range 4–7) to 2 (range 1–3) in Group B. Three patients required loperamide and six used dietary fiber modification (Table 3).

**Table 3:** Functional outcomes at last follow-up.

Outcome	Group A (*n* = 8)	Group B (*n* = 10)
Daily stools, median (range)	3 (2–4)	2 (1–3)
Fecal incontinence (Krickenbeck grade II)	2	0
Need for loperamide	2	1
Dietary fiber modification	3	3

## Discussion

Although staged management with initial ileostomy is the widely accepted approach for TCA, a subset of patients may present late yet remain clinically compensated. In our series, delayed referral and preserved clinical stability allowed safe single-stage definitive surgery without prior diversion. We acknowledge that this is not typical and should be reserved for carefully selected patients in experienced centers.

Our experience demonstrates that minimally invasive surgery (MIS) is a safe and effective strategy for managing total colonic aganglionosis (TCA). All procedures in our series were initiated laparoscopically and none required conversion to an open approach, reflecting the steady evolution surgical expertise of our surgical team. Although the study includes a comparison between laparoscopic Duhamel and laparoscopic Swenson procedures, the primary objective was not to establish the superiority of one technique over the other. Rather, this series reflects the progressive evolution of surgical expertise in the management of total colonic aganglionosis over time. Changes in operative strategy, technical refinements, and increasing familiarity with minimally invasive techniques influenced procedural selection and operative performance throughout the study period. Therefore, the comparison between both approaches should be interpreted within the context of the progressive evolution of surgical expertise and an evolving surgical practice rather than as a formal comparative effectiveness study.

With progressive accumulation of experience, several refinements in operative strategy and technical execution were introduced to optimize laparoscopic management. We did accurate identification of the transition zone by laparoscopic assessment of the entire bowel. In early cases, frozen-section biopsies were done only when no sizable funnel was detected or doubtful transitional zone was found but mandatory serial biopsies was taken to all later cases and became our standard to ensure reliable confirmation of ganglionated bowel prior to definitive pull-through.

The surgical approach to mobilization also evolved over time. Extensive mesenteric dissection, initially performed to facilitate bowel reach, was gradually replaced by a more selective vessel-preserving technique aimed at maintaining mesenteric perfusion and reducing tension on the pulled-through segment. To avoid orientation errors and mesenteric twisting during pull-through, intracorporeal marking sutures were later incorporated as a routine step.

Dealing with markedly dilated bowel was improved through optimization of port placement, improved traction strategies, and enhanced ergonomic positioning, atraumatic bowel handling which collectively improved visualization and dissection precision.

A more limited pelvic dissection was achieved through progressive accumulative surgeries,this minimize risk of the pelvic injury, use of energy device near mesenteric vessels was progressively restricted to preserve marginal vascularity. Standardization of operative steps, clear team role allocation, and continuous intraoperative review collectively contributed to improved efficiency contributed to improved efficiency and reduced operative times over the study period. Kauffman et al. (8) reported advantages of minimal invasive surgery as reduced surgical trauma, superior cosmetic results, faster postoperative recovery, and shorter hospital stay. The laparoscopic approach also allows seromuscular biopsies to detect level of aganglionic part before dissection. This minimizes the need for extensive transanal dissection and helps prevent twisting of the pulled-through ileum. We divided our cases into two groups. Group A underwent laparoscopic-assisted transanal Swenson-type ileoanal pull-through, whereas Group B underwent laparoscopic Duhamel surgery.

Our mean operative times (4 h for Group A and 5 h for Group B) compare favorably with those reported by Travassos et al. (9) (mean 6 h for laparoscopic Duhamel) and Cheung et al. (10) (mean 6 h 50 min for laparoscopic endorectal pull-through). This surgical efficiency was achieved through a well-defined surgical expertise and specific technical refinements implemented by our single team. Specifically, the routine use of a bipolar vessel-sealing device (LigaSure) significantly accelerated mesenteric devascularization and minimized intraoperative bleeding. Furthermore, standardizing port placements and establishing clear intraoperative role allocation allowed for ergonomic and seamless transitions between the laparoscopic and transanal phases.

Bischoff et al. (3) recommended mandatory ileostomy in all TCA cases. We instead conducted preoperative laparoscopic ileostomy only in neonates with obstruction or uncontrollable enterocolitis and proceeded with definitive pull-through earlier, delaying ileostomy closure until toilet training. For the 5 primary cases who presented without a prior stoma, a primary pull-through was performed without a protecting/diverting ileostomy. The rationale was that these patients were late referrals (around 3 years of age) who were exceptionally well-compensated clinically, showed acceptable growth, Meticulous intraoperative technique, using a linear GI stapler for a secure, tension-free anastomosis and utilizing energy devices (LigaSure) to preserve marginal vascularity, allowed for a safe single-stage approach without diversion in this carefully selected subgroup. Cheung et al. (10) reported primary laparoscopic endorectal pull-through in 10 TCA cases without stoma, similar to our five non-ileostomy patients (17).

We excluded patients with small-bowel aganglionosis beyond 10 cm, as this is associated with poorer functional outcomes and higher rates of fecal incontinence and soiling, as shown by Escobar et al. (11) and Laughlin et al. (12). Although Duhamel and Martin operations retain a short aganglionic segment that may improve stool consistency, Heath et al. (13) noted increased postoperative enterocolitis with this strategy. In our Group B, three patients experienced enterocolitis, but overall bowel function was satisfactory and continence outcomes were superior to Group A.

Go Miyano et al. (14) found no major advantage of laparoscopy vs. open surgery in the Duhamel procedure except for cosmetic benefit. We nevertheless prefer MIS for all TCA cases—whether Duhamel or Swenson—because it facilitates complete colonic dissection and provides excellent visualization even in laparoscopic-assisted pull-through after ileostomy, consistent with other laparoscopic Duhamel series (19, 20).

Our functional results show higher early stool frequency and soiling in Group A, gradually improving over follow-up, and a slightly higher rate of enterocolitis in Group B, consistent with the literature. Importantly, we observed no anastomotic leaks and no major early complications. Regarding functional outcomes, a notable disparity was observed: two patients in Group A (Swenson) developed Krickenbeck grade II fecal incontinence, whereas zero cases were reported in Group B (Duhamel). This clinical variance can be explained by the fundamental technical differences between the two procedures.

In Swenson approach,mobilization of the rectum was done circumferentially, this carries risk of injury of the pelvic nerve, disruption of the internal anal sphincter mechanism but in Duhamel approach, dissection of the distal aganglionic rectum was done only retrorectal, keeping inadvertent pelvic nerve intact, the Duhamel approach maintains better sphincter integrity and rectoanal reflex mechanisms,additional reservoir capacity contributing to superior continence outcomes in our cohort and remaining aganglionic part in Duhamel approach carries more risk of enterocolitis.

Limitations of this study include the small sample size and retrospective design. A larger, multicenter prospective study is needed to confirm these findings (18) and to refine patient selection criteria for each surgical technique.

## Conclusion

Laparoscopic management of total colonic aganglionosis is safe, feasible, and provides good long-term functional outcomes. Both laparoscopic Duhamel and Swenson-type pull-through techniques are effective; selection should be tailored to individual patient anatomy and continence risk.

## Data Availability

The raw data supporting the conclusions of this article will be made available by the authors, without undue reservation.

## References

[1] Hoehner JC Ein SH Shandling B Kim PCW. Long-term morbidity in total colonic aganglionosis. J Pediatr Surg. (1998) 33:961–5. 10.1016/S0022-3468(98)90515-2 9694078

[2] Ieiri S Suita S Nakatsuji T Akiyoshi J Taguchi T. Total colonic aganglionosis with or without small bowel involvement: a 30-year retrospective nationwide survey in Japan. J Pediatr Surg. (2008) 43:2226–30. 10.1016/j.jpedsurg.2008.08.049 19040940

[3] Bischoff A Levitt MA Peña A. Total colonic aganglionosis: a surgical challenge. How to avoid complications? Pediatr Surg Int. (2011) 27:1047–52. 10.1007/s00383-011-2960-y 21809028 PMC3175048

[4] Coran AG. A personal experience with 100 consecutive total colectomies and straight ileoanal endorectal pull-throughs for benign disease of the colon and rectum in children and adults. Ann Surg. (1990) 212:242–7. 10.1097/00000658-199009000-00002 2396880 PMC1358148

[5] Kimura K Nishijima E Muraji T Tsugawa C Matsumoto Y. A new surgical approach to extensive aganglionosis. J Pediatr Surg. (1981) 16:840–3. 10.1016/S0022-3468(81)80830-5 7338764

[6] Martin LW. Surgical management of total colonic aganglionosis. Ann Surg. (1972) 176:343–6. 10.1097/00000658-197209000-00010 5073220 PMC1355404

[7] Rintala RJ Lindahl HG. Proctocolectomy and J-pouch ileo-anal anastomosis in children. J Pediatr Surg. (2002) 37:66–70. 10.1053/jpsu.2002.29429 11781989

[8] Kauffman JD Snyder CW Danielson PD Chandler NM. 30-day Outcomes of laparoscopic versus open total proctocolectomy with ileoanal anastomosis in children and young adults: a combined analysis of the national surgical quality improvement project pediatric and adult databases. J Laparoendosc Adv Surg Tech A. (2019) 29:402–8. 10.1089/lap.2018.0576 30481105

[9] Travassos DV Bax NMA Van der Zee DC. Duhamel procedure: a comparative retrospective study between an open and a laparoscopic technique. Surg Endosc. (2007) 21(12):2163–5. 10.1007/s00464-007-9317-6 17483999 PMC2077356

[10] Cheung ST Tam YH Chong HM Chan KW Mou WC Sihoe DYJ. An 18-year experience in total colonic aganglionosis: from staged operations to primary laparoscopic endorectal pull-through. J Pediatr Surg. (2009) 44(12):2352–4. 10.1016/j.jpedsurg.2009.07.057 20006025

[11] Escobar MA Grosfeld JL West KW Scherer LR Rouse TM Engum SA, et al. Long-term outcomes in total colonic aganglionosis: a 32-year experience. J Pediatr Surg. (2005)40:955–61. 10.1016/j.jpedsurg.2005.03.043 15991177

[12] Laughlin DM Friedmacher F Puri P. Total colonic aganglionosis: a systematic review and meta-analysis of long-term clinical outcome. Pediatr Surg Int. (2012) 28:773–9. 10.1007/s00383-012-3117-3 22842648

[13] Heath AL Spitz L Milla PJ. The absorptive function of colonic aganglionic intestine: are the Duhamel and Martin procedures rational? J Pediatr Surg. (1985) 20:34–6. 10.1016/S0022-3468(85)80388-2 3973811

[14] Miyano G Ochi T Lane GJ Okazaki T Yamataka A. Factors affected by surgical technique when treating total colonic aganglionosis: laparoscopy-assisted versus open surgery. Pediatr Surg Int. (2013) 29:349–52. 10.1007/s00383-012-3247-7 23292536

[15] Marquez TT Acton RD Hess DJ Duval S Saltzman DA. Comprehensive review of procedures for total colonic aganglionosis. J Pediatr Surg. (2009) 44:257–65. 10.1016/j.jpedsurg.2008.10.055 19159753

[16] Bonnard A de Lagausie P Leclair MD Marwan K Languepin J Bruneau B. Definitive treatment of extended Hirschsprung's disease or total colonic form. Surg Endosc. (2001) 15:1301–4. 10.1007/s004640090092 11727138

[17] Yagi M Okuyama H Oue T Kubota A Ohyanagi H. One-stage laparoscopic pull-through operation without stoma for total colon aganglionosis. Pediatr Endosc Innov Techn. (2003) 7:169–73. 10.1089/109264103766757934

[18] Stenström P Kyrklund K Bräutigam M Engstrand Lilja H Stensrud K J Löf Granström A. Total Colonic Aganglionosis: Multicentre Study of Surgical Treatment and Patient-reported outcomes up to Adulthood. Wiley Online Library, (2020). 10.1002/bjs5.50317 PMC7528515 32658386

[19] Zhang X Cao G Tang S Chang X Li S Yang L. Laparoscopic-assisted Duhamel procedure with ex-anal rectal transection for total colonic aganglionosis. J Pediatr Surg. (2018) 53:531–6. 10.1016/j.jpedsurg.2017.06.009 28705638

[20] Xu WL LI SL Sun C Wang W Yang X Liu X. Single-site laparoscopic-assisted Martin-Duhamel procedure in the treatment of total colonic aganglion-osis with stage Ⅰ stoma and evaluation of postoperative defecation function. J Clin Ped Sur. (2020) 19:31–5. 10.3969/j.issn.1671-6353.2020.01.006

